# Case Report: A rare case of recurrent ascites after anti-Claudin18.2 antibody therapy for metastatic gastric cancer while responding sustainingly

**DOI:** 10.3389/fonc.2023.1211668

**Published:** 2023-08-23

**Authors:** Jinlu Liu, Dan Jiang, Qingqiang Lei, Qing Zhu, Hong Zhu

**Affiliations:** ^1^ Division of Abdominal Tumor Multimodality Treatment, Cancer Center, West China Hospital, Sichuan University, Chengdu, Sichuan, China; ^2^ Department of Pathology, West China Hospital Sichuan University, Chengdu, Sichuan, China

**Keywords:** gastric cancer, targeted therapy, monoclonal antibody, Claudin18.2, adverse effect

## Abstract

**Background:**

Gastric cancer remains one of the deadliest malignancies in the world, thus urgently requiring effective and safe therapeutics. Claudin18.2 is a member of the tight junction protein family specifically expressed in gastric cancer cells. Monoclonal antibodies targeting Claudin18.2 have been receiving increasing attention recently. ASKB589 is a humanized monoclonal antibody targeting Claudin18.2.

**Case presentation:**

This case described a 65-year-old Chinese man diagnosed with gastric cancer metastasizing to the liver and multiple lymph nodes. The biomarker examination revealed that he had proficient mismatch repair (pMMR), human epidermal growth factor receptor 2 (HER2) was negative, and the combined proportion score (CPS) of PD-L1 (22C3) was 1. After being proven to be moderately positive for Claudin18.2 expression, he received ASKB589 and CAPOX (oxaliplatin and capecitabine) chemotherapy. After a six-cycle therapy (from 14 July 2022 to 29 November 2022), the target tumor was evaluated for partial response (PR) by the investigator based on the enhanced CT scan according to the Response Evaluation Criteria in Solid Tumors (RECIST) 1.1 criteria. However, this patient also suffered from intolerable ascites that gradually aggravated during the therapy, which was not controlled well by the supporting therapy. Therefore, the patient stopped receiving the combined therapy in our hospital and did not receive any other anti-tumor treatment. After 4 months of discontinuation of the drug, the patient’s ascites almost disappeared, while the tumor continued to reduce and almost achieved clinically complete relapse (cCR). His progression-free survival (PFS) reached at least 10 months.

**Conclusion:**

This is the first case of severe ascites reported after anti-Claudin18.2 monoclonal antibody treatment for advanced gastric cancer. At the same time, the patient still benefited significantly from this incomplete treatment even after discontinuation of the drug and the PFS reached at least 10 months. The ascites might be an immune adverse effect related to the monoclonal antibody-induced antibody-dependent cytotoxicity (ADCC) and complement-dependent cytotoxicity (CDC). Further mechanisms remain to be investigated.

## Introduction

Gastric cancer is still one of the most incident and fatal malignant tumors of the digestive tract. Recently, the American Cancer Society released the latest cancer statistics: it is expected that 26,500 cases of stomach cancer will occur in the United States in 2023, with 11,130 deaths and a mortality rate of nearly 43% (1). Therefore, effective therapeutics are in urgent demand. Targeted therapy and immunotherapy have changed the treatment of advanced gastric cancer. The ToGA trial demonstrated that the addition of trastuzumab to the standard chemotherapy (oxaliplatin and capecitabine, CAPOX) could significantly prolong the overall survival (OS) in patients with HER2-positive advanced gastric cancer (13.8 versus 11.1 months) (2). Furthermore, results from a randomized phase 3 clinical trial chekmate649 showed that nivolumab combined with chemotherapy significantly prolonged progression-free survival (PFS) and OS in patients with advanced gastric cancer, especially those with PD-L1 CPS scores greater than or equal to 5 (3). However, for patients who lack the targets above, the prognosis is much worse. This has prompted researchers to constantly strive to discover new therapeutic targets.

Claudin18.2 is a member of the tight junction protein family on the cell membrane and exposes outside the membrane when cells transform to malignancy, rendering it accessible to targeted therapies (4, 5). Claudin18.2 has strictly limited expression in normal tissues but is abnormally activated in gastric cancer, and this abnormally activated Claudin18.2 molecule can be specifically targeted by monoclonal antibodies, making it a highly attractive target for anti-tumor therapy (6). The FAST study found that the addition of zolbetuximab (an anti-Claudin18.2 monoclonal antibody) resulted in longer PFS and OS than standard chemotherapy in advanced gastric/gastroesophageal adenocarcinoma patients with moderately or highly positive claudin18.2 expression (7). Here, we present a case of a 65-year-old man diagnosed with advanced gastric cancer diffusely metastasizing to the liver and multiple lymph nodes for the first time into our department. After a six-course combined therapy of ASKB589 (a humanized monoclonal antibody drug targeting Claudin18.2) and CAPOX chemotherapy, the patient’s tumor in the liver could hardly be detected by the enhanced CT scan. However, as treatment began, ascites gradually began to develop and progressively worsened, and eventually the patient had to stop the therapy. After 4 months of discontinuation, the ascites almost disappeared, while the tumor continued to reduce and almost achieved clinically complete relapse (cCR).

## Case presentation

**Figure 1:**
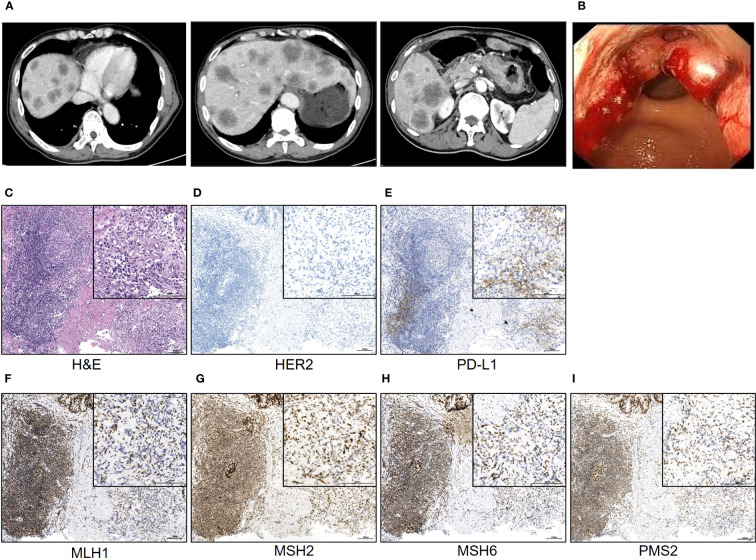
The typical image of the CT scan and gastroscopy examination. **(A)** The typical CT scan images; **(B)** the image of the gastroscopy; **(C–I)** the H&E staining and IHC staining of the neoplasm tissue of the patient. Scale bar = 100 μm.

A 65-year-old man was admitted to our hospital with recurrent intermittent stomachache in June 2022. He denied the symptoms of vomiting and nausea, fever, and loss of body weight. The physical examination (PE) did not show positive signs as well. Laboratory tests showed elevated levels of carcinoembryonic antigen (CEA, 271 ng/ml) and carbohydrate antigen 125 (CA125, 93.3 ng/ml). According to the symptom and the laboratory test results, malignancy was suspected. Therefore, he underwent a chest and abdomen computed tomography (CT) scan revealing an unevenly strengthened wall of the stomach body and multiple diffuse tumor metastases in the liver (**Figure 1A**). Further gastroscopy revealed an ulcerative neoplasm in the stomach body and the pathology examination demonstrated that it was adenocarcinoma (**Figure 1B**). Based on the above information, the patient was finally diagnosed with advanced gastric adenocarcinoma metastasizing to multiple abdominal lymph nodes and liver.

Because of the late stage of this patient, surgery was not recommended. Subsequently, the following therapy-related targets were detected: MLH1, MSH2, MSH6, and PMS2 were positive; CPS of PD-L1 (clone number 22C3) staining was 1; and HER2 staining was negative (**Figures 1C–I**). The standard first-line regimen was chemotherapy including CAPOX (oxaliplatin and capecitabine), SOX (oxaliplatin and S-1), or FLOT (oxaliplatin, fluorouracil and docetaxel). Because this patient and his family have expressed a desire to participate in clinical trials, he was screened and then included in a clinical trial titled “Phase I/II Clinical Study of the Safety, Tolerability, Pharmacokinetics and Antitumor Activity of ASKB589 Injection in Patients with Locally Advanced or Metastatic Solid Tumors” (NCT04632108) and received the CAPOX chemotherapy and ASKB589 combination therapy subsequently. ASKB589 is a monoclonal antibody drug targeting Claudin18.2 to induce ADCC and CDC to remove the tumor provided by ASK-Pharm Ltd. (China). The expression of Claudin18.2 in this patient was moderately positive, as detected by immunohistochemistry (IHC) staining. The regimen was as follows: ASKB589 (6 mg/kg) and oxaliplatin (130 mg/m^2^) were intravenously dripped on day 1, capecitabine (1,000 mg/m^2^) was orally infused twice a day from day 1 to day 14, and the above therapeutics were repeated every 3 weeks.

**Figure 2:**
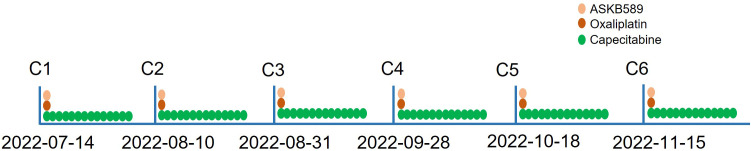
The treatment scheme of the combined therapy of ASKB589 and CAPOX chemotherapy.

**Figure 3:**
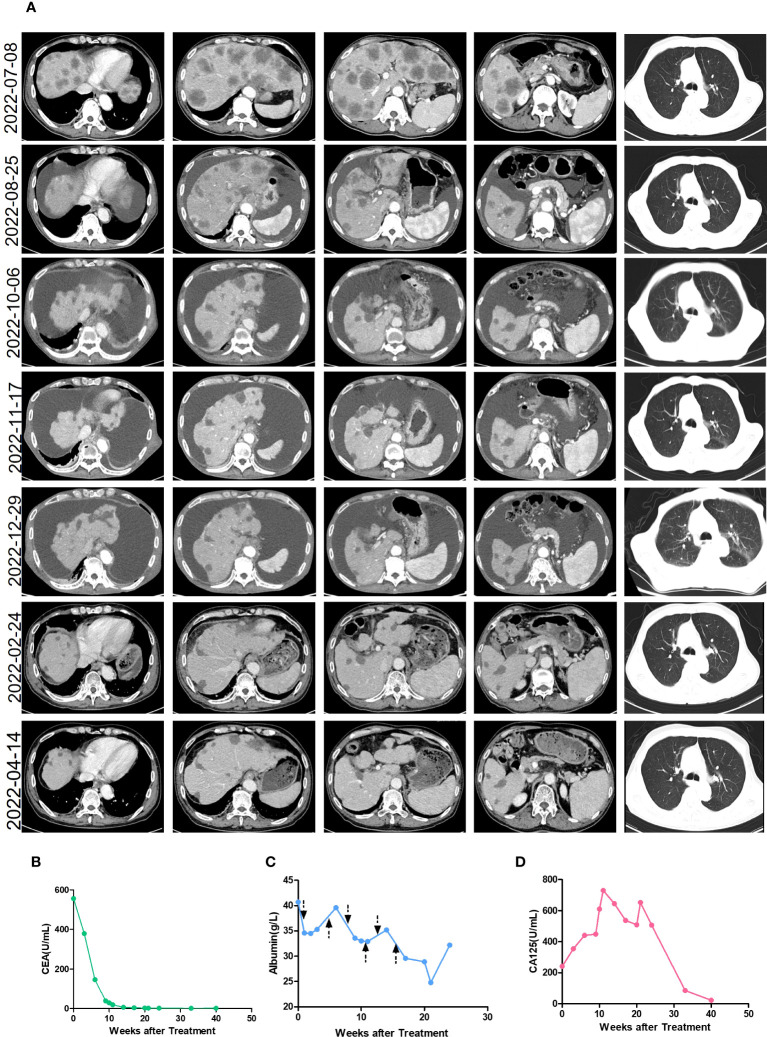
The clinical characteristics during treatment. **(A)** The enhanced CT images of the patient during treatment. The treatment began on 14 July 2022 and stopped on 29 November 2022. **(B)** The level of CEA during the treatment. The treatment began in week 0 and stopped in week 20. **(C)** The level of albumin during the treatment. The treatment began in week 0 and stopped in week 20. The arrow refers to the time point of the ASKB589 infusion. **(D)** The level of CA125 during the treatment. The treatment began in week 0 and stopped in week 20.

He received six courses of ASKB589 and chemotherapy in total from July 2022 to November 2022 (**Figure 2**). CT scan revealed a satisfactory response (PR, almost cCR) with the liver masses almost disappearing and CEA decreased to the normal level (**Figures 3A, B**). However, he developed peritoneal effusion and hypoalbuminemia after 5 weeks of the treatment (**Figures 3A, C**). Meanwhile, prior to the start of therapy, he was found with proteinuria (1+), but blood creatinine and glomerular filtration rate (GFR) remained normal. As treatment continued, the patient developed a much larger volume of ascites and CA125 grew significantly, while there was no significant change in albumin and urine protein (**Figure 3**). He was then treated supportively with paracentesis, human albumin, and diuretic drugs. Pathologic biopsy of the extracted ascites revealed no evidence of tumor cells but mainly lymphocytes. The laboratory examination showed a serum-ascites albumin gradient (SAAG) >11 g/L, total protein 6.4 g/L, lactate dehydrogenase (LDH) 56 IU/L, and adenosine amino hydrolase (ADA) 1.8 IU/L in the ascites. However, the ascites was not relieved significantly after the supportive treatment above. Therefore, he had to stop the anti-tumor therapy in December 2022 and did not receive other anti-tumor treatments like radiotherapy, chemotherapy, or immunotherapy anymore. Surprisingly, the ascites almost disappeared after the discontinuation and the CT images revealed a sustained reduction of masses in the liver even in April 2023 (more than 4 months after stopping the drug). This is a case where a patient developed large-volume recurrent ascites with concurrent regression of liver metastasis.

## Discussion

Gastric cancer remains one of the leading causes of cancer-related deaths worldwide, with more than 1.3 million deaths in 2020 (13% of all cancer deaths) (8). For patients with advanced stomach cancer who have no therapeutic target (e.g., Microsatellite Instability High, MSI-H, HER2 positive, CPS score≥5), the current treatment benefit is limited (9). Based on data from preclinical models and clinical samples, the anti-Claudin18.2 antibody emerged as a novel target therapy for stomach cancer (4, 6, 10–12). ASKB589 is a humanized monoclonal antibody targeting Claudin18.2 on the surface of gastric cancer cells, which can accurately recognize and bind to the claudin18.2 epitope of tumor cells, and through ADCC and CDC to remove the tumor. In this case, the combination therapy of ASKB589 and CAPOX chemotherapy significantly reduced the liver metastases and persisted for 4 months after the discontinuation of the drug (PFS at least 10 months), which is not common in advanced stomach cancer patients. The standard of first-line treatment for patients with unresectable advanced or recurrent gastric cancer is fluoropyrimidine and platinum-based chemotherapy, and the median PFS and median OS for these patients are 4.9 to 6.0 months and 10.5 to 14.1 months, respectively (13). Anti-claudin18.2 therapy in addition to chemotherapy can further increase the anti-tumor efficacy. Recently, the Glow clinical trial also reported the prolonging OS and PFS of zolbetuximab combined with CAPOX chemotherapy compared to chemotherapy alone (14). Anti-Claudin18.2 antibodies combined with standard chemotherapy is likely to be a potential first-line treatment for advanced patients.

Ascites, the pathological accumulation of fluid in the abdominal cavity, usually occurs repeatedly in the condition of tumor peritoneal metastasis in GC patients (15). However, ascites biopsy of this case revealed no evidence of malignant cells and laboratory examination demonstrated ascites as a transudate with SAAG >11 g/L, total protein 6.4 g/L, LDH 56 IU/L, and ADA 1.8 IU/L, which was not in accordance with metastatic peritoneal malignancies. Meanwhile, the presence and remission of ascites in this patient were closely related to the treatment, thus indicating that ascites may be an adverse effect of the anti-tumor therapy. Adverse reactions of chemotherapy mainly include adverse reactions at the injection site (phlebitis), digestive system adverse reactions (nausea and vomiting), bone marrow suppression, organ function damage (toxicity of heart, liver, and kidney), neurotoxicity, hair loss, and skin reactions while the adverse effects of monoclonal antibody-targeted therapy are hypersensitive reaction, arterial and venous thromboembolic events, diarrhea, nausea, vomiting, and so on (16–18). As for anti-Claudin18.2 antibody therapy, the most common adverse effects are vomiting and nausea (19). Interestingly, this patient developed recurrent massive ascites without significant evidence of liver or kidney dysfunction and peritoneal metastasis. Moreover, the level of albumin decrease cannot explain the large amount of effusion. Randy et al. reported a similar case of a urothelial carcinoma developing massive ascites after the nivolumab therapy while the tumor had been reduced. After the flow cytometry analysis of the ascites, they found a high percentage of PD-1^+^ CD8^+^ and HLA-DR^+^ CD8^+^ T cells there. Therefore, they speculated that the ascites were secondary to non-specific immune-related adverse events such as autoimmune peritonitis (20). In this case, we also suspected that the recurrent ascites with tumor reduction may be a result of the monoclonal antibody-related immune adverse effects (irAEs).

## Conclusion

In conclusion, we reported a Chinese man’s case that can be easily misdiagnosed as progressing even after anti-tumor therapy because of his increasing ascites. After six courses of treatment of the combined therapy of ASKB-589 and chemotherapy, he had a good response but developed intolerable recurrent ascites and finally stopped the therapy mentioned above. However, even 4 months after the discontinuation, he still responded to the treatment and the PFS reached as long as 10 months. It indicated that it is critical to differentially diagnose the reason for ascites to avoid misdiagnosing.

## Data availability statement

The original contributions presented in the study are included in the article/supplementary material. Further inquiries can be directed to the corresponding author.

## Ethics statement

Written informed consent was obtained from the individual for the publication of this case report and any potentially identifiable images or data included in this article.

## Author contributions

JL and HZ conceived and wrote the manuscript. DJ carried out the pathological examination of this case. DJ and QL collected the pathological images of this case. JL, HZ, DJ, QL and QZ revised the manuscript. All authors contributed to the article and approved the submitted version.

## References

[1] Siegel RL Miller KD Wagle NS Jemal A. Cancer statistics, 2023. CA Cancer J Clin (2023) 73(1):17–48. doi: 10.3322/caac.21763 36633525

[2] Bang YJ Van Cutsem E Feyereislova A Chung HC Shen L Sawaki A. Trastuzumab in combination with chemotherapy versus chemotherapy alone for treatment of HER2-positive advanced gastric or gastro-oesophageal junction cancer (ToGA): a phase 3, open-label, randomised controlled trial. Lancet (2010) 376(9742):687–97. doi: 10.1016/S0140-6736(10)61121-X 20728210

[3] Shitara K Ajani JA Moehler M Garrido M Gallardo C Shen L. Nivolumab plus chemotherapy or ipilimumab in gastro-oesophageal cancer. Nature (2022) 603(7903):942–8. doi: 10.1038/s41586-022-04508-4 PMC8967713 35322232

[4] Singh P Toom S Huang Y. Anti-claudin 18.2 antibody as new targeted therapy for advanced gastric cancer. J Hematol Oncol (2017) 10(1):105. doi: 10.1186/s13045-017-0473-4 28494772 PMC5427576

[5] Türeci Ö Mitnacht-Kraus R Wöll S Yamada T Sahin U. Characterization of zolbetuximab in pancreatic cancer models. Oncoimmunology (2019) 8(1):e1523096. doi: 10.1080/2162402X.2018.1523096 30546962 PMC6287799

[6] Sahin U Koslowski M Dhaene K Usener D Brandenburg G Seitz G. Claudin-18 splice variant 2 is a pan-cancer target suitable for therapeutic antibody development. Clin Cancer Res (2008) 14(23):7624–34. doi: 10.1158/1078-0432.CCR-08-1547 19047087

[7] Sahin U Türeci Ö Manikhas G Lordick F Rusyn A Vynnychenko I. FAST: a randomised phase II study of zolbetuximab (IMAB362) plus EOX versus EOX alone for first-line treatment of advanced CLDN18.2-positive gastric and gastro-oesophageal adenocarcinoma. Ann Oncol (2021) 32(5):609–19. doi: 10.1016/j.annonc.2021.02.005 33610734

[8] Sung H Ferlay J Siegel RL Laversanne M Soerjomataram I Jemal A. Global cancer statistics 2020: GLOBOCAN estimates of incidence and mortality worldwide for 36 cancers in 185 countries. CA Cancer J Clin (2021) 71(3):209–49. doi: 10.3322/caac.21660 33538338

[9] Lavacchi D Fancelli S Buttitta E Vannini G Guidolin A Winchler C. Perioperative tailored treatments for gastric cancer: times are changing. Int J Mol Sci (2023) 24(5). doi: 10.3390/ijms24054877 PMC10003389 36902306

[10] Klamp T Schumacher J Huber G Kühne C Meissner U Selmi A. Highly specific auto-antibodies against claudin-18 isoform 2 induced by a chimeric HBcAg virus-like particle vaccine kill tumor cells and inhibit the growth of lung metastases. Cancer Res (2011) 71(2):516–27. doi: 10.1158/0008-5472.CAN-10-2292 21224362

[11] Yao F Kausalya JP Sia YY Teo AS Lee WH Ong AG. Recurrent fusion genes in gastric cancer: CLDN18-ARHGAP26 induces loss of epithelial integrity. Cell Rep (2015) 12(2):272–85. doi: 10.1016/j.celrep.2015.06.020 26146084

[12] Al-Batran SE Schuler M Zvirbule Z Manikhas G Lordick F Tureci O. IMAB362: a novel immunotherapeutic antibody targeting the tight-junction protein component CLAUDIN18.2 in gastric cancer. Ann Oncol (2016) 27:141–2. doi: 10.1093/annonc/mdw237.06

[13] Kang YK Chen LT Ryu MH Oh DY Oh SC Chung HC. Nivolumab plus chemotherapy versus placebo plus chemotherapy in patients with HER2-negative, untreated, unresectable advanced or recurrent gastric or gastro-oesophageal junction cancer (ATTRACTION-4): a randomised, multicentre, double-blind, placebo-controlled, phase 3 trial. Lancet Oncol (2022) 23(2):234–47. doi: 10.1016/S1470-2045(21)00692-6 35030335

[14] Shah MA Ajani JA Al-Batran S-E Bang Y-J Catenacci DVT Enzinger PC. Zolbetuximab plus CAPOX versus CAPOX in first-line treatment of claudin18.2(+)/HER2(-) advanced/metastatic gastric or gastroesophageal junction adenocarcinoma: GLOW phase 3 study. J Clin Oncol (2022) 40(4). doi: 10.1200/JCO.2022.40.4_suppl.TPS365

[15] Imamoto H Oba K Sakamoto J Iishi H Narahara H Yumiba T. Assessing clinical benefit response in the treatment of gastric Malignant ascites with non-measurable lesions: a multicenter phase II trial of paclitaxel for Malignant ascites secondary to advanced/recurrent gastric cancer. Gastric Cancer (2011) 14(1):81–90. doi: 10.1007/s10120-011-0016-6 21327925

[16] Nevins S McLoughlin CD Oliveros A Stein JB Rashid MA Hou Y. Nanotechnology approaches for prevention and treatment of chemotherapy-induced neurotoxicity, neuropathy, and cardiomyopathy in breast and ovarian cancer survivors. Small (2023):e2300744. doi: 10.1002/smll.202300744 37058079 PMC10576016

[17] Pilkington K Wieland LS Teng L Jin XY Storey D Liu JP. Coriolus (Trametes) versicolor mushroom to reduce adverse effects from chemotherapy or radiotherapy in people with colorectal cancer. Cochrane Database Systematic Rev (2022) 11). doi: 10.1002/14651858.CD012053.pub2 PMC9707730 36445793

[18] Hansel TT Kropshofer H Singer T Mitchell JA George AJ. The safety and side effects of monoclonal antibodies. Nat Rev Drug Discov (2010) 9(4):325–38. doi: 10.1038/nrd3003 20305665

[19] Tureci O Sahin U Schulze-Bergkamen H Zvirbule Z Lordick F Koeberle D. A multicentre, phase IIa study of zolbetuximab as a single agent in patients with recurrent or refractory advanced adenocarcinoma of the stomach or lower oesophagus: the MONO study. Ann Oncol (2019) 30(9):1487–95. doi: 10.1093/annonc/mdz199 PMC6771222 31240302

[20] Sweis RF Zha Y Pass L Heiss B Chongsuwat T Luke JJ. Pseudoprogression manifesting as recurrent ascites with anti-PD-1 immunotherapy in urothelial bladder cancer. J Immunother Cancer (2018) 6(1):24. doi: 10.1186/s40425-018-0334-x 29618376 PMC5883337

